# Can clubfoot scoring systems predict the number of casts and future recurrences in patients undergoing Ponseti method?

**DOI:** 10.1186/s13018-021-02261-4

**Published:** 2021-04-05

**Authors:** Mohammad Ali Tahririan, Mohammadreza Piri Ardakani, Sara Kheiri

**Affiliations:** Department of Orthopaedic Surgery, Isfahan University of Medical Sciences, Isfahan, Iran

**Keywords:** Clubfoot, Pirani score, Dimeglio score, Ponseti method, Recurrence, Number of casts

## Abstract

**Background:**

Congenital clubfoot is one of the common congenital orthopaedic deformities. Pirani and Dimeglio scoring systems are two classification systems for measuring the severity of the clubfoot. However, the relation between the initial amount of each of these scores and the treatment parameters is controversial.

**Methods:**

Patients with severe and very severe idiopathic clubfoot undergoing Ponseti treatment were entered. Their initial Pirani and Dimeglio scores, the number of castings as a short-term treatment parameter, and the recurrences as a long-term parameter until the age of three were prospectively documented.

**Results:**

One hundred patients (143 feet) with mean age of 9.51 ± 2.3 days including 68 males and 32 females and the mean initial Pirani score of 5.5 ± 0.5 and the mean initial Dimeglio score of 17.1 ± 1.6 were studied. The incidence of relapse was 8.4 %( *n* = 12). The mean initial Pirani score (*P* < 0.001) and the mean initial Dimeglio score (*P* < 0.003) of the feet with recurrence were significantly more than the non-recurrence feet. The mean number of casts in the recurrence group (7 ± 0.9) was significantly more than the feet without recurrences (6.01 ± 1.04) (*P* = 0.002). The ROC curve suggested the Pirani score of 5.75 and the Dimeglio score of 17.5 as the cut-off points of these scores for recurrence prediction.

**Conclusion:**

In our study, Pirani and Dimeglio scores are markedly related with more number of casts and recurrence in patients with severe and very severe clubfoot. Also, we have introduced new cut-off points for both classification systems for prediction of recurrence. To the best of our knowledge, this finding has not been introduced into the English literature.

## Introduction

Idiopathic talipes equinovarus or clubfoot is a common congenital orthopaedic deformity [1]. The severity of the deformity could be classified by Pirani scoring and Dimeglio scoring systems [2, 3]. While the two scoring systems measure different aspects of clubfoot, they seem to be correlated and complementary [4]. Regardless of the initial severity, Ponseti method has become the gold standard treatment of choice for clubfoot [5]. The technique consists of frequent weekly manipulation and casting followed by an Achilles tenotomy and brace wearing until the walking age [6].

In practice, the casting period and the recurrence of the deformity are among the common concerns of the parents. However, there are controversies about the correlation between these two parameters and the initial severity of the deformity measured by Dimeglio and Pirani scoring systems [4, 5, 7–9].

It has been suggested that the different severities of the clubfoot could be differently correlated with the number of casts [3–5]. Therefore, we decided to study only on patients with severe or very severe clubfoot based on the Dimeglio classification system.

Here in this study, we evaluated the effect of the initial Pirani score and the Dimeglio score on the number of casts as the short-term parameter and the recurrence of clubfoot as the long-term parameter of the Ponseti method. Also, we have sought for mathematical equations between each grading systems and the number of casts and investigated if there could be any cut-off points for each score to predict the risk of recurrence accurately.

## Materials and methods

The study was performed in line with the principles of the declaration of Helsinki. Approval was granted by the Ethics Committee of the university. The patients with idiopathic clubfoot who were admitted to our orthopaedic university medical centre between 2014 and 2017 were prospectively studied. Patients whose parents did not sign the consent form, who were previously treated in other centres, who were older than 15 days of age in the first admission, the ones with non-idiopathic cases, patients with other simultaneous deformities, and also the cases with mild or moderate severities based on the Dimeglio grading system after the first admission were excluded. Finally, 100 patients (143 feet), 32 females and 68 males, with the mean age of 9.51 ± 2.3 days were included in the study.

**Fig. 1:**
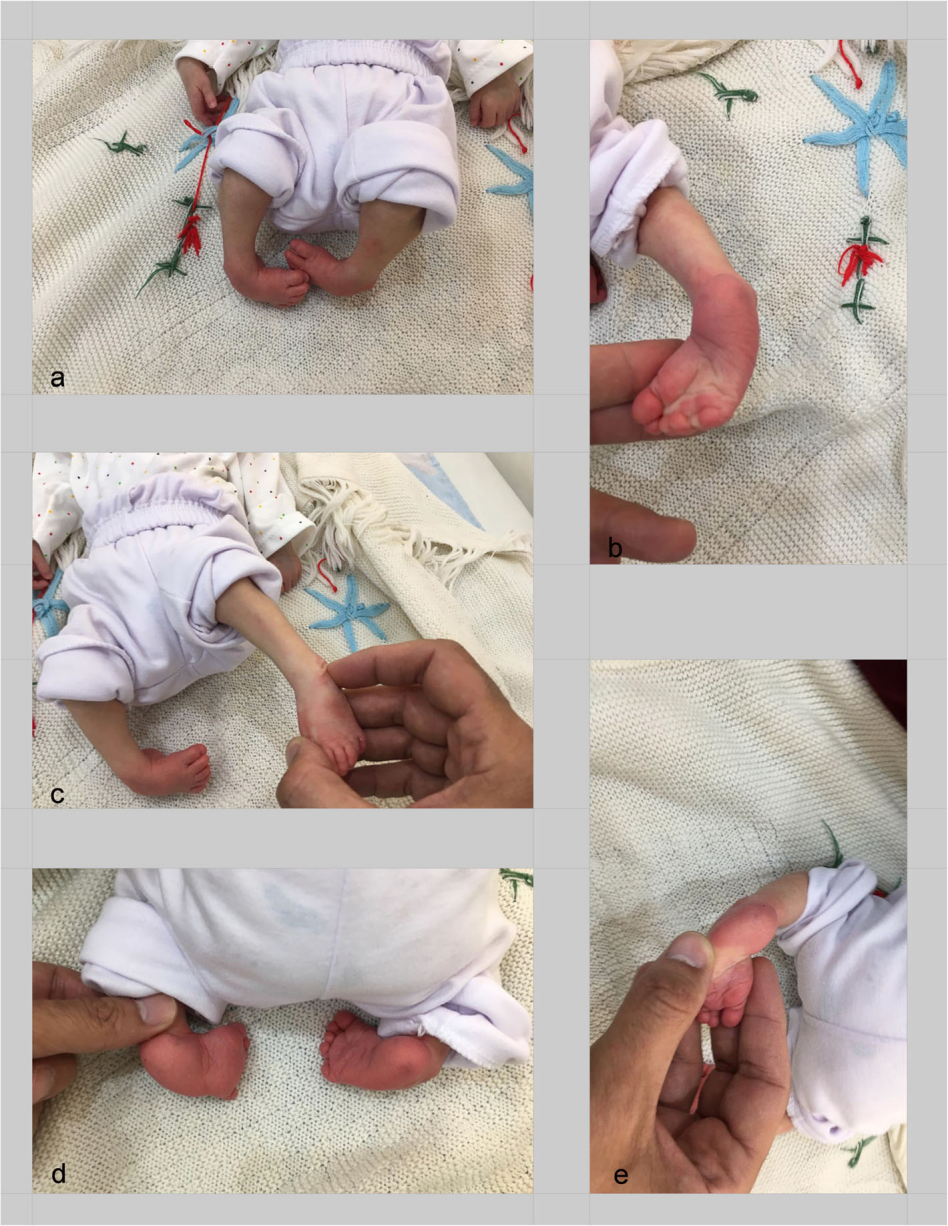
Pirani and Dimeglio score were measured in all the patients before the start of the treatment. Notice to the curved lateral border (**a**), medial crease (**b**), lateral head of talus (**c**), empty heel and medial crease (**d**), and posterior crease (**d**), which determine Pirani score. Also, equinus, varus, rotation, adduction, cavus, and muscle abnormality are other prominent factors for determining the Dimeglio score which could be found in the photo

All the patients were treated by one highly skilled attending orthopaedic surgeon based on the Ponseti treatment protocol. The initial Pirani score and Dimeglio score were measured in the first admission (Fig. 1).

**Fig. 2:**
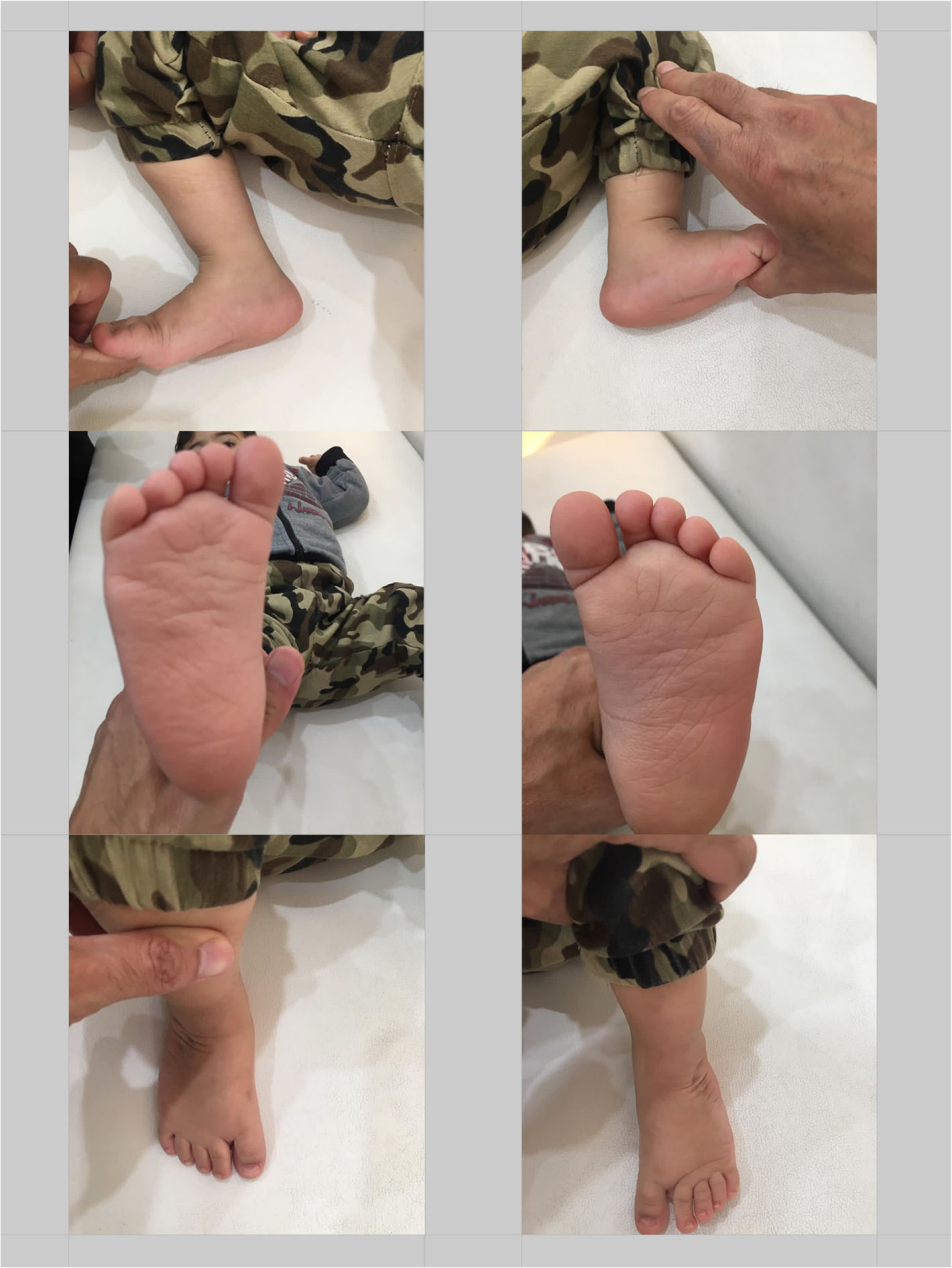
Photos of the same patient with clubfeet in 1-year follow-up sessions after tenotomy. Note how the feet appearance has improved compared with pre-treatment shape shown in Fig. 1

All the patients went through frequent weekly manipulation and casting until the deformity was partially corrected based on obtaining 70 degrees of abduction and also the clinical judgement of the orthopaedic surgeon. The number of casts until the percutaneous Achilles tenotomy for each patient was written. Achilles tenotomy was done for all the cases followed by one more casting for 3 weeks. Then, Denis-Browne brace was administered for 23 h a day for the first 3 months, then 20 h a day for the next 3 months and then 16 h a day for the third 3 months and finally 12 h a day till the walking age. Then, the patients wore reverse last shoes during the day and wore the Denis-Browne brace during sleep. All the patients were followed up every 3 months (Fig.2), while using the positive communication style for them in which the parent's understanding of the importance of correct brace wearing and the early signs of recurrence was evaluated in a non-judgmental manner followed by a positive affirmation of the parents until 3 years of age (between 2017 and 2020).

Relapse criteria included dynamic supination of the forefoot, heel varus, and metatarsal adductus. For the relapse cases, repeated manipulation and casting along with modification surgery was done. One skilled orthopaedic surgeon did all the procedures and data recordings to minimize the probable biases.

SPSS version 22 was used to analyse the data. Pearson correlation, independent *T* test, Fisher’s exact test, analysis of covariance (ANCOVA) test, and receiver operating characteristic (ROC) curves were run to analyse and depict the data.

The correlation coefficients between 0 and 0.2 were considered as having no correlation, 0.2–0.4 were regarded as low correlation, 0.4 to 0.6 were considered as moderate correlation, 0.6 to 0.79 were regarded as a marked correlation, and 0.8 to 1 were considered as high correlation [10].

*P* value less than 0.05 was obtained to quantify the statistical significance of each test.

## Results

One hundred patients (143 feet), 32 females and 68 males, were included in the study. The mean age of the patients was 9.51 ± 2.3 days. The incidence of recurrence was 8.4 %( *n* = 12), while 91.6% of the feet (*n* = 131) did not have any criteria for the recurrence.

The mean initial Pirani score of all feet was 5.5 ± 0.5, while the mean initial Dimeglio score was 17.1 ± 1.6.

Independent *t*-test showed that the mean initial Pirani score (*P* < 0.001) and the mean initial Dimeglio score (*P* < 0.003) of the feet with recurrence were significantly higher than the non-recurrence feet (Table 1).

**Table 1:** Association between relapse and mean initial Pirani score and mean initial Dimeglio score

	Recurrence		Non-recurrence		*p*-value
	mean	SD	mean	SD	
Initial Pirani score	5.9	0.1	5.4	0.5	0.001
Initial Dimeglio score	18.3	1.5	16.9	1.5	0.003

The difference between mean age in the patients who experienced recurrence (8.9 ± 1.8) and the mean age in the patients who did not experience recurrence (9.5 ± 2.4) was shown by independent *t*-test to be not significant (*P* = 0.39). Therefore, in our study, mean age was not significantly different between recurrence and the non-recurrence group.

Three of the female patients’ feet (6.1% of the feet) experienced recurrence while 9 of the male patients’ feet (9.6% of the feet) experienced recurrence. Fisher’s exact test showed the difference between them is not significant (*P* = 0.75). Therefore, in our study, recurrence occurrence was not significantly different in different genders.

Also, ANCOVA test showed that there was a significant difference in the initial Pirani score between recurrence group and the non-recurrence group (*P* < 0.00084) and also in the initial Dimeglio score between recurrence group and the non-recurrence group (*P* < 0.0031) after being adjusted for age and gender. Means that age and gender did not compromised the effect of initial scorings on the recurrence of the clubfoot.

The mean number of casts was 7 ± 0.9 in the feet who had recurrence and 6.01 ± 1.04 in the feet who did not experience any recurrences. Independent *t*-test showed that their difference is statistically significant. (*P* = 0.002). It means that in our study, the patients without recurrence needed statistically significant fewer number of casts than the ones with recurrence.

**Fig. 3:**
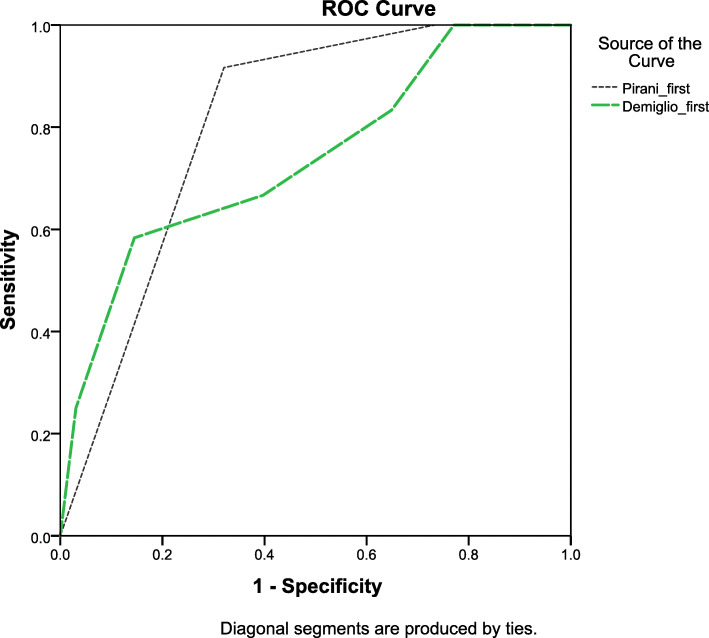
ROC curve depicting the chance of recurrence based on initial Dimeglio and Pirani scores

According to the ROC curve shown in Fig. 3, the cut-off point for the Pirani score for predicting the recurrence of clubfoot is 5.75, with 91.7% sensitivity and 67.9% specificity. In other words, this cut-off point could predict 91.7% of the recurrences (as a long-term parameter) and 67.9% of the non-recurrence cases correctly.

Also, the cut-off point for predicting the recurrence based on the Dimeglio score was calculated as 17.5, with 66.7% sensitivity and 61.3% specificity. This means that with this cut-off point, 66.7 % of the recurrence cases and 61.3% of the non-recurrence cases could be accurately predicted.

Pearson correlation showed that mathematically there is a direct linear relationship between the number of casts and the initial Pirani score (*r* = 0.653, *P* < 0.001) and the linear regression equation between the number of casts (*Y*) based on the initial Pirani score (*x*) is as follows:

$$ Y=-1.01+1.30x $$

Therefore, in most of our cases, the number of casts (as a short-term parameter of treatment result) could be predicted using this equation based on the initial Pirani score in the patients’ first admissions.

Pearson correlation showed that mathematically there is a direct linear relationship between the number of casts and the initial Dimeglio score (*r* = 0.749, *P* < 0.001) and the linear regression equation between the number of casts (*Y*) and the initial Dimeglio score(*x*) is as follows:

$$ Y=-2.71+0.52x $$

Therefore, in most of our cases, the number of casts could be predicted by using this equation based on the initial Dimeglio score in the patients’ first admissions.

## Discussion

Idiopathic clubfoot is a common congenital orthopaedic deformity worldwide [1]. Ponseti method was introduced as the gold standard method of choice for clubfoot treatment [6]. It consists of frequent weekly manipulation and casting followed by a percutaneous Achilles tenotomy (if needed) and wearing the proper brace afterward [6]. Dimeglio and Pirani classification systems have been introduced as two valid classification systems in clubfoot with good inter-observer and intra-observer coefficients [5, 11].

Dimeglio scoring is based on the foot reducibility; while the Pirani scoring system is based on the morphology of the foot, and considering their correlation and complementarity, it is suggested that both scores be reported on the first admission [4]. Dimeglio scoring has 20 scores in total, and it is classified as benign if the score is less than 5, moderate if the score is between five and ten, severe if the score is between ten and 15, and very severe if the final score is between 15 and 20 [2]. In our study, we only entered patients with severe or very severe clubfeet as it is reported that the outcomes of Ponseti method could be different in patients with different severities [5].

However, the strength of these two scores in predicting the short-term and long-term parameters of the Ponseti treatment has remained controversial [5, 12]. In this study, we encountered the number of casts as a short-term course of treatment parameter while the recurrence was used as a long-term factor.

As it has been reported that the number of casts could be influenced by the skill of the surgeon, all the patients’ course of treatments were done only by one highly skilled attending orthopaedic surgeon for preventing any probable operator’s bias [12].

Lampasi et al. studied on the patients with clubfoot who were less than 3 months old and had initial Pirani score more than two or initial Dimeglio score more than 6. They reported that both initial scores have moderate to good reliability and accuracy for predicting the number of casts, and they both have collinear relations for predicting the number of casts [12].

Chu et al. studied on patients with clubfoot with different severities. They reported a low correlation between the total number of casts till the initiation of foot abduction orthosis and the two initial scoring systems [7]. However, in this study, we only entered patients with severe or very severe clubfoot who went under tenotomy. Also, we did not count the last cast after tenotomy, like most of the other studies’ methods [5, 8, 13], and as there were other studies suggesting no significant correlation between the number of casts and the initial scores when taking the post-tenotomy cast into account [14, 15]. The difference in their results could be because the tenotomy was not done for all their cases. Therefore, the post-tenotomy cast was not counted for all. Both of the initial scores in our results showed marked correlations with the number of casts.

Agarwal et al. also found a direct linear relationship between the initial Pirani score and the number of casts but with a different regression equation from ours. Their suggested equation of the number of casts and the initial Pirani score was:

Number of casts = 4.1 + 0.6 × initial Pirani score (*r*^2^ = 0.05; multiple *r* = 0.24; *P* < 0.001) [16].

As seen, our equation has a greater *r*^2^ value (0.426) than theirs, which could suggest a better power for predicting the number of casts.

The difference between the two suggested equations could be explained by the different inclusion criteria of two studies. In Agarwal et al.’s study, patients with idiopathic clubfoot who were up to 10 years of age were included. Their results showed that, compared with age, the effect of initial Pirani score on the number of casts was ten times more predictable [16]. However, due to the controversial reports of the effect of age on the results of the Ponseti method, we only included neonates less than 15 days old [9, 17–19].

In Fan et al.’s study, patients with mild to very severe clubfoot were entered, and a quadratic or cubic relation between the number of casts and the initial Pirani or Dimeglio scores was reported [5]. But to the best of our knowledge, there was no study suggesting an equation of the initial Dimeglio score and the number of casts (as this study has mentioned), while having both formulas at the same time can help the orthopaedic surgeon to have a better estimation of the duration of casting period (which is a very common concern among patients’ parents).

Multiple factors have been proposed for evaluating the long-term effects of treatment [20, 21]. Muffuli et al. studied on 14 patients with the mean age of 19.1 ± 2.3 and tested anthropometric variables as well as functional assessments including active and passive dorsiflexion and single leg hopping ability along with subjective functional parameters for them in two separate visits (1 week and 1 month apart and twice on each appointment). They proposed that an assisted administered functional questionnaire in addition to the passive and active dorsiflexion measurements could help with a valid long-term treatment result prediction [21]. The suggested criteria for long-term results, although novel, were not useful for our study due to the very young age of the patients.

Also, Chesney et al. used a patient reported subjective assessment of outcomes as well as objective outcomes including calf circumference, length of the foot, and the range of motion at the ankle for evaluating the long-term results of patients with clubfoot. They reported that there is a correlation between these anthropometric outcomes and the subjective outcomes. And therefore by using the mentioned objective outcomes, the subjective outcomes could also be anticipated [20]. Their method, although very interesting in considering patients evaluation of the treatment along with anthropometric data, was not executable for our study due to the young age of the patients. However, we have evaluated some of these parameters indirectly by measuring the Dimeglio and Pirani scores.

Therefore, considering such limitations, we decided to choose the recurrence status as our long-term treatment outcome. Recurrence, as a long-term parameter, seems to have a controversial relationship with initial Pirani and Dimeglio scores [8, 22, 23].

In Fan et al.’s study, although the number of casts was higher in the group with recurrence, the initial Dimeglio score was lower in this group [5]. Our data does not support this finding according to the suggested direct linear relationship between the initial Dimeglio score and the number of casts with a positive slope.

In our results, we mentioned that according to the ROC curves, an initial Pirani score of 5.75 could predict 91.7% of the recurrences and 67.9% of the non-recurrence cases correctly. However, based on the Pirani scoring system, the Pirani score could take any number from 0 to 6 with the smallest possible fraction of half a full point, but not a quarter or third or any other fractions. Therefore, we suggest using 5.5 as the cut-off point for recurrence prediction instead of 5.75, which was mentioned in our results.

Also, it was shown that an initial Dimeglio sore of 17.5 could predict 66.7 % of the recurrences and 61.3% of the non-recurrence cases accurately. However, based on the Dimeglio scoring system, the Dimeglio score could take any whole number from 0 to 20 [2]. Therefore, we suggest using 18 as the cut-off point of the initial Dimeglio score for recurrence prediction instead of 17.5.

To the best of our knowledge, there was no other study in English literature suggesting such cut-off points for recurrence prediction.

According to our results, the cut-off point for the initial Pirani score seems to be more sensitive in predicting the recurrence cases compared with the cut-off point for the initial Dimeglio score. This is a unique outcome as it has been suggested that the Dimeglio score has a better predictive value than the Pirani score as it includes more parameters [12].

Fan et al. reported that the Pirani score could measure the deformity more severely than the Dimeglio score. This might be because of measurement bias due to more parameters included in the Dimeglio scoring system, as well as the different natures of the two scoring systems [5]. This could explain our finding that the initial Pirani score of the foot predicted the recurrence more accurately than initial Dimeglio score as it probably reported the initial deformity more severely.

However, as shown, both scores do not have enough strength in estimating the non-recurrence chance fully, and this emphasizes the need for other foot parameters to be included in future proposed scoring systems.

Clubfoot’s recurrence has been reported to be highly correlated with compliance for brace wearing [15, 22, 23], which is also reported to be related to the severity of the deformity, as patients with a more severe deformity tend to wear brace less often than patients with milder deformities due to its inconvenience for fitting in [24]. We tried to decrease the effects of these confounding factors by entering only patients with severe or very severe clubfeet into our study and also strictly following them up every 3 months while using a more positive communication style, which has been suggested to decrease the rate of recurrence [25]. However, due to the lack of sensors within our casts, the exact identification of compliant brace-wearing patients was impossible. Also, considering that recurrences might occur at any time till the end of the growth, we have probably lost the recurrences which happened after the age of 3. We also did not measure patients’ weights and foot length in our study and we suggest future studies evaluate these variables as potential predictive factors for the treatment outcomes.

Also, we hypothesized that the calf volume might be a predictive factor for the recurrence of clubfoot, as it could indirectly affect the gait pattern and weight bearing of the patients in the future. Barker et al. reported a reliable method in order to measure the calf volume in patients with clubfoot [26]. However, we were not able to provide such detailed, protected condition as mentioned in their study in order to report the patients’ calf volume. Also, as all patients were neonates, we could not measure the calf volume in active standing condition. We suggest that future studies evaluate the effect of calf volume on the recurrence as well as Pirani and Dimeglio score changes in a controlled condition. Table 2 depicts the limitations and also the advantages of our study in summary.

**Table 2:** Limitations versus advantages of the study

Limitations	Advantages
Lack of sensors in the casts to measure the compliance of patients in brace wearing	Regular follow up of the patients for two years while using a positive communication style in order to decrease the chance of recurrence
Lack of more than 2 years long term follow up of the patients	Showing that both classification systems are related to the recurrence of the deformity
Lack of the data of the weight of the patients and their foot length and calf volumes	Reporting cut-off points for initial Pirani and Dimeglio scores for the prediction of the recurrence (Which is not reported in the literature so far)
	Reporting a higher sensitivity for initial Pirani score than the initial Dimeglio score for prediction of the recurrence of the deformity
	Showing that both initial Pirani and Dimeglio scores are related to the number of cast for modifying the deformity (and therefore an estimation of the duration of casting period)
	Inclusion of patients with the same age and severity in order to decrease the possible confounding factors
	Reporting a mathematical formula between the number of casts and the initial Pirani score (With a higher r^2 than other formulas reported before)
	Reporting a mathematical formula between the number of casts and the initial Dimeglio score for the first time in English literature

## Conclusion

Initial Pirani and Dimeglio scores seem to be able to predict the required number of casts for modifying the deformity (as the short-term outcome which could also help the orthopaedic surgeon to have an estimation about the casting period) and the recurrence of the deformity (as the long-term outcome) of the patients with severe and very severe clubfeet. However, further studies with longer follow-up durations and in more controlled conditions as well as considering other possible predicting factors of clubfoot treatment including sensor utilization in the casts, measuring feet’s length, and calf volume are encouraged.

## Data Availability

The datasets generated and/or analysed during the current study are not publicly available due to patients’ privacy but are available from the corresponding author on reasonable request.
